# Myeloma: A Lot of Progress, Still a Long Way to Go

**DOI:** 10.3390/cancers13236087

**Published:** 2021-12-02

**Authors:** Gábor Mikala, Gergely Varga

**Affiliations:** 1 Department of Hematology and Stem Cell Transplantation, South Pest Central Hospital, National Institute for Haematology and Infectious Diseases, 1097 Budapest, Hungary; 2 Department of Internal Medicine and Haematology, Semmelweis University, 1085 Budapest, Hungary; vargager@gmail.com

It was Bart Barlogie who made a clear point by stating in one of his lectures that any myeloma that is not cured will eventually turn into a resistant disease with aggressive clinical behaviour.

Still, a better understanding of the molecular mechanisms of aggressive tumour biology may help to expose the Achilles’ heel of the disease. Certainly, the aberrant three-dimensional organization of the telomeres may be a marker of genomic instability and consequential disease aggressiveness, as described by Rangel-Pozzo and co-workers [1]. Short and dysfunctional telomeres with a reduced ability to bind protective shelterin proteins can lead to “uncapped” telomeres that activate the DNA damage response and may drive genomic instability, leading to aggressive tumour biology. In their paper, distinctive 3D telomeric profiles are shown to correlate with disease aggressiveness and response to certain myeloma therapies. Moreover, the behaviour of smouldering myeloma can be stratified according to the telomere aggregate intensity and numbers.

Cancer drug resistance and biological aggressivity may not be understood exclusively through an investigation of the cellular processes of the tumour cells. Genomic instability, aberrant DNA damage repair, apoptosis inhibition, altered metabolomics, and activation of drug-excluding transporters are all important determinants of anticancer drug activity and drug resistance. Recently, it has become well accepted that individual cells may no longer be considered the fundamental units of cell biology. In a publication by Matula and co-workers [2], evidence is provided about the role of mitochondrial exchange between myeloma cells and their neighbouring stromal cells. From co-culture studies, data have emerged that show myeloma cells rapidly respond to the presence of anti-myeloma drugs by acquiring mitochondria from non-malignant stromal cells and delivering “damaged” mitochondria in exchange. Tunnelling nanotubes and partial cell fusion seem to be the major underlining mechanisms. As a consequence of mitochondrial exchange, tumour cell survival and ATP production increase, while superoxide levels decrease. Interestingly, a hint of the differential effects of daratumumab and isatuximab, two anti-CD38 therapeutic antibodies with different binding epitope locations on their target molecule, are noted in this paper with respect to mitochondrial exchange.

Immunomodulatory drugs (IMiDs) are oral anti-myeloma drugs, pioneered by thalidomide, and have been the mainstay of myeloma therapy since 1999. In a review by Charlinski and co-workers [3], detailed insight is provided on their mode of action through the modulation of cereblon ubiquitin ligase activity, the main target of all IMiDs. Though highly similar in chemical structure, IMiDs differ in not only their anti-myeloma activity, but their side effect profile, too. Importantly, the antitumor activity of these compounds is at least three pronged: both the direct inhibition of plasma cell survival, and the modulation of stromal cell support of myeloma growth, as well as an increase in the anti-tumour immune response, are involved. These effects are all detailed in this review and provide an explanation of the clinical activity of both IMiDs and the new group of thalidomide analogs, CRBN E3 ligase modulators (CELMoDs).

While IMiDs paved the way for immunotherapy of multiple myeloma, targeted antibody therapies have recently entered routine clinical use; antibody-drug-conjugates (ADCs) represent a relatively new platform. ADCs and bispecific antibodies—designed to bring T cells within striking distance to target tumour cells—are all discussed as promising new avenues of immune therapy by Ackley and co-workers [4]. In their review, each class of immune therapy is covered, and the paper importantly points to CAR-T cell therapies as one of the most promising therapeutic alternatives for myeloma at the present time.

An entire review by Martino and co-workers [5] is dedicated to recent advances in CAR-T cell technology applied to multiple myeloma. CAR-T cells represent an exciting single infusion approach to provide continuous antitumor therapy for myeloma patients. B-cell maturation antigen (BCMA)-directed CAR-T cell technology has shown remarkable efficacy in multiple myeloma due to the universal and exclusive expression of BCMA on the surface of mature B-cells and plasma cells. Importantly, the expression of BCMA is thought to be essential for normal plasma cell survival. Unfortunately, the present CAR-T approaches do not offer a universal response, and most treated patients, even those who reached deep remissions, eventually relapse. This review critically details the most important open questions of the time in this field, such as the lack of CAR-T cell persistence, antigenic loss, and also, the much-needed improvement of safety following CAR-T administration.

While CAR-T cells, at present, may seem to be the most promising single therapy in myeloma, one should not forget the entire picture of the immune system and the role it plays in myeloma. This aspect is richly addressed in a review by Krejcik and co-workers [6]. Immune dysfunctions may be involved in the transformation from MGUS to multiple myeloma and its re-engagement may be a way to regain control of the disease. This review provides a deep understanding of not just the immune system (mainly T cells and NK cells) in myeloma but also its reactivation using immunotherapeutic modalities such as vaccination and allogeneic stem cell transplantation techniques. CAR-T and CAR-NK cell strategies, using genetically altered lymphocytes, are also positioned within this framework. A glimpse on oncolytic virotherapy and on the still-controversial use of immune checkpoint inhibitors is also offered.

Due to myeloma therapy with novel agents, the survival of our patients has dramatically improved. In fact, this great improvement is aligned with a higher percentage of treated myeloma patients reaching complete remission, though from early on it has been evident that not all complete remissions are created equal. Minimal residual disease (MRD) emerged as a feasible new paradigm to be reached and followed, as is vividly discussed in a review by Bravo-Perez and co-workers [7]. MRD is currently defined as having one malignant cell in at least 10^5^ normal cells of bone marrow, and this has become an important landmark in myeloma therapy as superior PFS and OS were observed in patients who achieved MRD negativity. MRD results that can be obtained using multiparametric flow cytometry (MFC) and high throughput next-generation sequencing (NGS) are reviewed, and the advantages, as well as drawbacks of each methodology, are discussed. Since plasma cell infiltration in myeloma may be patchy, imaging techniques (PET/CT and MRI) also entered the field of establishing MRD negativity, and their importance is especially emphasized in the case when extramedullary infiltration is suspected. The authors point out that MRD evaluation is destined to soon guide clinicians in their choice of optimal therapeutic strategies.

High-dose therapy followed by autologous stem cell transplantation (ASCT) is a standard of care in multiple myeloma. As myeloma that reaches daratumumab-refractoriness is usually a very aggressive and resistant form of the disease, the successful application of salvage ASCT as a rescue approach is of great significance. In a paper by Yarlagadda and co-workers [8], the results of such therapy for 69 consecutive patients are presented. They report an impressive 80% response rate with a PFS of 7.2 months and an OS of 19.3 months, an outcome better than most of the currently published alternative rescue therapy results. Moreover, salvage ASCT showed a remarkable ability to correct cytopenias in this heavily pre-treated patient population. This is of critical importance as salvage ASCT—applied as a bridging therapy—may aid patients in enrolling in potentially life-saving clinical trials of novel drugs.

Renal failure is an important feature, with presentation in up to 50% of myeloma cases. Bachmann and co-workers [9] review three protocols utilized in two large national trials by the German Multiple Myeloma Study Group with PI- and IMiD-based triplets. The trials excluded patients with a GFR below 30 mL/min, but still accepted many patients with moderate renal failure, giving an opportunity to look into the effect of these protocols on the amelioration of renal function. The three protocols were cyclophosphamide-bortezomib-dexamethasone, bortezomib-lenalidomide-dexamethasone (VRD), and lenalidomide-adriamycin-dexamethasone. Interestingly, the patients who received bortezomib-lenalidomide-dexamethasone had a higher risk of worse renal function following induction. This paper emphasizes the importance of a rapid haematological response helping to reach renal recovery as well as the importance of avoiding toxicities. With regard to these, in patients with renal failure, modern protocols free from potentially nephrotoxic drugs such as daratumumab-bortezomib-thalidomide-dexamethasone might be preferable over lenalidomide-based triplets.

Skeletal events are a hallmark of multiple myeloma and used to almost invariably signal the end stage of this disease, leading to distorting fractures and immobility. The introduction of anti-bone-resorption therapy was, therefore, a tremendous step forward in reducing the frequency and severity of this complication; however, it is not without its own side-effects. Medication-related osteonecrosis of the jaw (MRONJ) is an uncommon but important adverse reaction to these drugs. Due to the improving survival, the level of exposure to these drugs becomes longer, and we might expect to see more such complications. Therefore, prevention and early intervention is important, as Beaumont and co-workers [10] emphasize in their paper. The risk of MRONJ is higher in malignancy than in non-malignant osteoporosis. The main drugs causatively associated with MRONJ are bisphosphonates and denosumab; however, the role of others, including IMiDs and corticosteroids, are discussed too. Dental procedures are triggering factors in the majority of cases, with dental extraction being responsible for two-thirds of MRONJs, but poor oral health and a pre-existing periodontal or periapical infection was also indicated as a risk factor. Therefore, the most important measure to reduce MRONJ is a comprehensive dental examination, and for a definitive solution of any problems to be suggested before starting bisphosphonates or denosumab.

The eleventh paper in this edition discusses medical treatments of multiple myeloma from the viewpoint of the payers. The cost of cancer treatment in Europe has quadrupled over the last two decades, partially driven by an aging population and increasing cancer incidence, but mostly by the cost of novel drugs. The costs related to myeloma care are among the highest. Seefat and co-workers [11] analysed 13 published cost analyses, mostly of comparisons of new triplets—including daratumumab, carfilzomib, pomalidomide, elotuzumab, ixazomib, and panobinostat—with the lenalidomide- and bortezomib-based doublets utilized as backbones in most of the triplet protocols. They found that the cost-effectiveness ratios of these novel protocols are usually above the current willingness-to-pay thresholds. This paper reminds us that we cannot forget about the cost of treatments, especially with the arrival of a new wave of even more expensive cellular and non-cellular immuno-therapeutic strategies.

The introduction of novel and characteristically myeloma-specific agents has revolutionized the clinical therapeutic efficacy seen in this devastating disease. On the other hand, the steady emergence of resistance to even the most promising tumour-cell-specific drugs has become an ever-increasing problem. Innovative therapeutics targeting not (just) the tumour cells but rather the bone marrow microenvironment and the immune system may provide an avenue to reach more efficacious myeloma therapy and an eventual cure for this disease. In this Special Issue, the papers highlight multiple important aspects of the research and development that have recently occurred in the field of multiple myeloma and deepen our understanding of the emerging myeloma therapeutics.
